# Brain-Derived Neurotropic Factor, Vascular Endothelial Growth Factor and Matrix Metalloproteinases as Markers of Metabolic Status in Non-Growth Hormone-Treated Girls With Turner Syndrome

**DOI:** 10.3389/fendo.2021.722199

**Published:** 2021-08-27

**Authors:** Ewa Błaszczyk, Jakub Gawlik, Joanna Gieburowska, Agnieszka Tokarska, Małgorzata Kimsa-Furdzik, Grzegorz Hibner, Tomasz Francuz, Aneta Monika Gawlik

**Affiliations:** ^1^Department of Pediatrics and Pediatric Endocrinology, Faculty of Medical Sciences in Katowice, Medical University of Silesia, Katowice, Poland; ^2^Student Scientific Society, Department of Biophysics, Jagiellonian University Medical College, Kraków, Poland; ^3^Department of Biochemistry, Faculty of Medical Sciences in Katowice, Medical University of Silesia, Katowice, Poland

**Keywords:** Turner syndrome, metabolic status, MMP-1/matrix metalloproteinase-1, MMP-2/matrix metalloproteinase-2, MMP-9/matrix metalloproteinase-9, BDNF/brain-derived neurotrophic factor, GDNF/glial cell line-derived neurotrophic factor, VEGF/vascular endothelial growth factor

## Abstract

**Background:**

Turner syndrome (TS) presents a high risk of congenital heart defects and may predispose to both obesity and related metabolic complications. Hence the search for new markers as potential early predictors of the metabolic syndrome (MetS) and cardiovascular diseases appears warranted.

**Objective:**

To assess MMP-1 (matrix metalloproteinase-1), MMP-2 (matrix metalloproteinase-2), MMP-9 (matrix metallopeptidase-9), BDNF (brain-derived neurotrophic factor), GDNF (glial cell line-derived neurotrophic factor), and VEGF (vascular endothelial growth factor) in non-MetS TS girls not treated with growth hormone (GH) *vs*. healthy short stature girls, and to assess the connection with basic metabolic parameters.

**Method:**

The concentrations of circulating MMP-1, MMP-2, MMP-9, BDNF, GDNF and VEGF were measured in 12 patients with TS not treated with growth hormone. The control group was composed of 17 girls with non-pathologic short stature. The patients’ clinical and biochemical phenotypes were determined by weight, height, total cholesterol, HDL cholesterol, triglycerides, glucose, aminotransferases, IGF1, TSH and fT4.

**Results:**

There were no differences in mean age, weight, BMI Z-Score, or hSDS between the studied group and the controls; however, they differed in baseline values of ALT (18.2 ± 4.2 *vs*. 14.2 ± 4.1, p= 0.02), BDNF [29951.5 (26176.9 – 41271.9) *vs*. 23131.7 (18392.4 – 28313.3), p=0.01] and MMP-2 [91.8 (71.7 – 111.0) *vs*. 143.6 (123.7 - 244.5), p< 0.001]. BDNF correlated with ALT activity (r = 0.56 p = 0.002) and BMI Z-score (r = 0.38 p = 0.042), while MMP-2 correlated with HDL concentration (r = 0.48 p = 0.029) in all the patients. The analysis of the study group alone revealed significant positive correlations between MMP-9 and TSH (r = 0.74 p = 0.036), BDNF and both ALT (r = 0.73 p = 0.038) and TSH (r = 0.85 p = 0.008), and a negative correlation between MMP-1 and fT4 (r = -0.75 p = 0.032). The control group did not present any significant correlations.

**Conclusion:**

The higher concentrations of BDNF and lower of MMP-2 found in girls with TS without MetS compared to healthy girls with short stature, could have a major impact on the future “natural” development of the metabolic status. Our findings need further studies.

## Introduction

Obesity and other components of the metabolic syndrome (MetS), such as hypertension, dyslipidemia and type 2 diabetes that occur in childhood, have been identified as significant determinants of cardiovascular diseases in adulthood (CVD) (1). Many studies indicate that metabolic disorders occur more frequently in the Turner Syndrome (TS) population and are noticeable as early as in childhood (2, 3). Additionally, heart defects, mainly aortic coarctation and bicuspid aortic valve, are more common in Turner syndrome (2). All these factors cause shorter life expectancy in the group of TS patients (4). It remains unclear whether cardiometabolic risks in TS are a consequence of intrinsic factors or the result of modifiable metabolic risk factors. Thus, the search for new markers as potential early predictors of the natural development of metabolic disorders/complications seems to be justified. Our previous pilot study regarded selected metabolic markers in TS girls undergoing growth hormone (GH) therapy (5). In this study, we re-examine brain-derived neurotrophic factor (BDNF), glial cell line-derived neurotrophic factors (GDNF), vascular endothelial growth factor (VEGF) and matrix metalloproteinases (MMPs) in a newly assembled group of patients, this time before the onset of GH-therapy (to exclude its potential influence) with a view to investigate their possible connection with basic metabolic parameters.

BDNF is a member of the nerve growth factor family, which plays an important role in brain metabolic and feeding regulation (6). BDNF affects glucose, lipid and energy metabolism and is considered an anorexigenic factor (7). Its concentration correlates negatively with body weight (8) and age (9) and is believed to be a factor for cardioprotection, neuroprotection and aging. Furthermore, patients diagnosed with MetS or acute coronary syndrome (10) have significantly lower plasma BDNF levels. These reports support the metabotropic deficit hypothesis (11), according to which, the absence of neurotrophins can predispose to the development of metabolic diseases. There is paucity of research on the role and plasma concentration of BDNF in patients with TS. However, in one study, BDNF plasma concentrations were proven to be significantly higher in the group of adult TS patients compared to the control group (12). Our pilot study also confirmed higher BDNF levels in girls with TS (5).

GDNF plays a neuroprotective role for neurons that participate in the regulation of energy intake and expenditure (13). The study on transgenic mice with overexpression of GDNF in glial cells revealed that they are protected from obesity, glucose intolerance, and resistance to insulin caused by high-fat meals (14). Like BDNF, GDNF seems to have a role in the suggested metabotropic hypothesis of metabolic disorders.

VEGF appears to have a metabolic effect, too. In obese people, the development of blood vessels is insufficient and lower concentrations of VEGF are observed, which results in hypoxia of adipocytes and local inflammation (15). Hypoxia is known to cause glucose intolerance (16), whilst ongoing low-grade inflammation leads to insulin insensitivity (17). However, the published data regarding the relationship between VEGF and metabolic processes are inconclusive. One study demonstrated a positive correlation between the concentrations of circulating VEGF levels and BMI in healthy male subjects (18), whilst another proved increased levels of VEGF in overweight and obese individuals, with higher levels in females (19). To date, no large studies on VEGF in TS in the context of obesity and carbohydrate disorders have been published. Our previous pilot work showed no differences in VEGF concentrations between TS girls treated with GH and short stature girls (5).

Matrix metalloproteinases (MMPs) are a group of enzymes that degrade extracellular matrix components with expression regulated by growth factors or ongoing inflammation (20). MMP-1 seems to be involved in the development of adipose tissue (21), while plasma levels of MMP-2 and MMP-9 are elevated in patients with the metabolic syndrome (22). In our previous study, MMP-1 was significantly higher in the study group, with a significant positive correlation with Z-score BMI; we found no differences in the levels of other MMPs (5).

## Subjects and Methods

Twenty-nine patients were enrolled in this prospective study. The study group included 12 patients with Turner syndrome (TS), confirmed by karyotyping with routine G-banding according to the recommendations of the American College of Medical Genetics. The control group consisted of 17 girls with nonpathological/non-syndromic short stature (healthy, short stature girls, with normal karyotype, normal birth weight [non-SGA] and excluded endocrine causes of short stature). We have extended the control group compared to the pilot study (5).

### Clinical Phenotype of Study Participants

The detailed anthropometrical analysis was based on weight and height measurements, along with body mass index (BMI) calculation, using the standard formula of weight (kg) divided by height (m) squared. Weight was measured with a Seca scale with a precision of 100 g, and height with a Harpenden stadiometer with a graduation of 0.1 cm. A BMI above the 97th percentile was classified as obesity, while a BMI between the 90th and 97th percentile as overweight based on the BMI chart for healthy girls (23). Height was expressed as standardized values (height standard deviation score (hSDS) based on Turner Syndrome Growth Chart by Ranke (24) and on the Polish growth chart for healthy girls (23). hSDS was calculated using the following formula: hSDS = child’s height − height for 50 pc/0.5 ∗ (height 50 pc − height 3 pc). Based on the age, sex, BMI, and appropriate reference standard, the BMI Z-score was calculated using the international (International Obesity Task Force; IOTF) body mass index (BMI) cut-offs (25). The patients’ bone age was determined based on the X-ray of the non-dominant hand using the Greulich- Pyle Atlas (26). The Tanner scale was used for puberty assessment (27). TS girls underwent cardiological evaluation to assess heart defects and detect hypertension before the onset of GH-therapy.

### Biochemical Phenotype of Study Participants

Morning fasting venous blood samples were collected to measure MMP-1 (matrix metalloproteinase-1), MMP-2 (matrix metalloproteinase-2), MMP-9 (matrix metallopeptidase-9), BDNF (brain-derived neurotrophic factor), GDNF (glial cell line derived neurotrophic factor), and VEGF (vascular endothelial growth factor). The concentrations of these markers were determined with sandwich ELISA, using kits distributed by the R&D systems.

Concentrations of total cholesterol (TCh), HDL cholesterol (HDL-chol) and triglycerides (TG) were analyzed enzymatically (Beckman Coulter, Brea, CA). An enzymatic test (hexokinase method) was used for the quantitative determination of glucose (Beckman Coulter). In order to assess the concentrations of TG, HDL-chol and fasting glucose in children aged 3 to 11 years old, the results were categorized into 3 groups according to the IDEFICS study (28): 0 - good result; 1 - observation necessary; 2 - intervention required. For older children, IDF percentile charts were used - values were classified as 0 (good result) or 1 (incorrect result) (29).

The concentrations of fT4 (free thyroxine), TSH (thyroid-stimulating hormone), ALT (alanine transaminase), AST (aspartate transaminase), and IGF-1 (insulin-like growth factor 1) were also determined. Serum concentrations of fT4 and TSH were measured with a chemiluminescent immunometric assay (IMMULITE 2000 Free T4 and IMMULITE 2000 Third Generation TSH, respectively; Siemens). Alanine and aspartate aminotransferase activity in the serum was assessed according to the International Federation in Clinical Chemistry (Beckman Coulter).

### Statistical Analysis

Data processing and statistical analyses were performed using Statistica 13.3 PL software. p value < 0.05 was considered significant. Variables with a normal distribution were analyzed using T-tests and reported as mean ± standard deviation (SD), those without normal distribution were analyzed using the Mann–Whitney U test and reported as median with interquartile range.

The study was conducted according to the Declaration of Helsinki and approved by the Ethics Committee of the Medical University of Silesia [resolution number KNW/0022/KB1/162/15/16)]. Informed consent was obtained from each participant aged over 16, a parent, or a legal guardian.

## Results

The clinical characteristics of all participants are presented in **Table 1**. The groups did not differ in chronological or bone age. There were no differences in mean weight, BMI Z-Score, or hSDS. Obesity was diagnosed in one patient in the study group, and overweight in two. No excess body weight was observed in the control group patients. A statistically significant difference in birth weight was found between the control group and the study group. According to the Tanner scale, 5 controls and 2 study patients started spontaneous puberty (no higher than Tanner stage B2). Heart defects were observed in 3 patients in the study group: coarctation of the aorta (1 patient) and bicuspid aortic valve (2 patients), whilst 3 patients in this group had confirmed hypertension.

**Table 1:** Clinical characteristics of the study and control groups.

	Study group (n = 12)	Control group (n = 17)	P value
Age	8.9 ± 3.5	10.1 ± 2.5	NS
Bone age [years]	7.6 ± 3.8	8.2 ± 2.6	NS
BMI Z-Score	0.18 ± 1.41	-0.63 ± 0.71	NS
hSDST	0.99 ± 1.06	0.95 ± 0.80	NS
hSDS	-2.57 ± 0.83	-2.82 ± 0.53	NS
Birth Weight [g]	2847.5 ± 431.3	3275.6 ± 338.3	0.01

T-test. Data are presented as mean ± SD. hSDS, height standard deviation score; hSDSTS, hSDS according to growth chart for Turner syndrome; BMI Z-score, body mass index Z-score; NS, not significant.

The biochemical characteristics of both groups are presented in **Table 2**. Two patients in the study group had impaired fasting glucose, one hypertriglyceridemia and three hypercholesterolemia. In the control group, two girls had hypertriglyceridemia and one hypercholesterolemia. None of the patients had lower HDL concentrations. The analyzed groups differed in mean baseline values of ALT, which, although within normal ranges, were significantly higher in the study group.

**Table 2:** Biochemical characteristics of the study and control groups.

	Study group (n = 12)	Control group (n = 17)	P value
Tch [mg/dl]	186.9 ± 26.4	177.9 ± 20.5	NS
LDL [mg/dl]	118.0 ± 20.0	101.2 ± 22.0	NS
HDL [mg/dl]	53.5 ± 8.5	61.6 ± 10.5	NS
TG [mg/dl]	76.7 ± 31.7	75.8 ± 23.1	NS
fT4 [ng/dl]	1.45 ± 0.26	1.34 ± 0.13	NS
TSH [IU/ml]*	2.99 (2.71 – 3.33)	2.325 (1.84 – 3.00)	NS
ALT [IU/l]	18.2 ± 4.2	14.2 ± 4.1	0.02
AST [IU/l]	29.9 ± 7.1	31.8 ± 5.2	NS
glucose`0 [mg/dl]	88.1 ± 7.6	86.1 ± 6.3	NS
IGF1 [ng/ml]	178.3 ± 89.5	138.3 ± 57.7	NS

T-test. Data are presented as mean ± SD. * Mann–Whitney U test. Data presented as median with interquartile range. TCh, total cholesterol, HDL, HDL cholesterol, TG, triglycerides, fT4, free T4; TSH, thyroid-stimulating hormone; ALT, alanine transaminase; AST, aspartate transaminase; IGF-1, insulin-like growth factor 1; NS, not significant.

The concentrations of the proposed markers in the two groups are shown in **Table 3**. No differences were found in VEGF, MMP-9, and MMP-1 concentrations, while BDNF and MMP-2 were significantly different in the two groups. GDNF concentrations were below the limit of detection.

**Table 3:** Markers’ concentrations in the study and control groups.

	Study group (n = 12)	Control group (n = 17)	p-value
VEGF [pg/ml]	15.99 (6.19 – 22.72)	48.11 (13.93 - 92.48)	NS
MMP-9 [ng/ml]	165.41 (97.36 – 385.00)	227.96 (193.87 – 380.19)	NS
BDNF [pg/ml]	29951.54 (26176.87 – 41271.88)	23131.69 (18392.37 – 28313.33)	0.01
MMP-1 [pg/ml]	2078.14 (1408.12 – 2539.28)	1489.91 (992.25 – 2495.51)	NS
MMP-2 [ng/ml]	91.84 (71.71 – 111.03)	143.63 (123.67 - 244.46)	<0.001

Mann–Whitney U test. Data presented as median with interquartile range. MMP-1, matrix metalloproteinase-1; MMP-2, matrix metalloproteinase-2; MMP-9, matrix metallopeptidase 9; BDNF, brain-derived neurotrophic factor; VEGF, vascular endothelial growth factor; NS, not significant.

**Figure 1:**
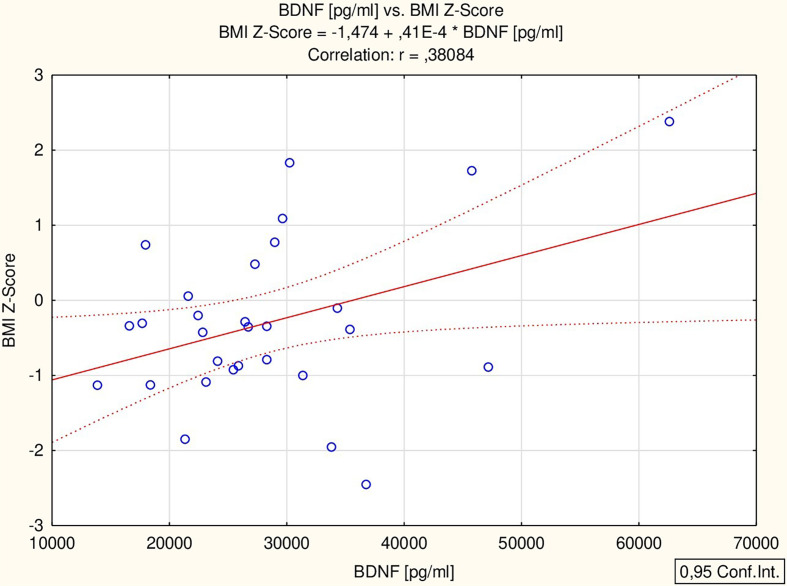
Correlation of BDNF with BMI Z-score; body mass index Z-score; BDNF, brain-derived neurotrophic factor.

**Figure 2:**
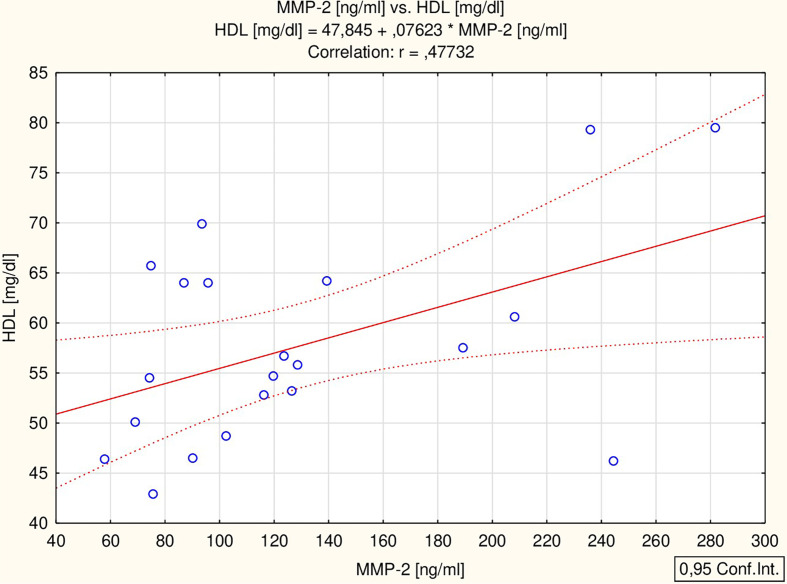
Correlation of MMP-2 with HDL. MMP-2, matrix metalloproteinase-2; HDL, HDL cholesterol.

All the proposed markers were correlated to glucose, total cholesterol, HDL-chol, TG, ALT, IGF-1, TSH and ft4 concentrations, as well as BMI Z-score. BDNF correlated with ALT activity (r = 0.56 p = 0.002) and BMI Z-score (r = 0.38 p = 0.042) (**Figure 1**), while MMP-2 correlated with HDL concentration (r = 0.48 p = 0.029) (**Figure 2**) in all the patients. The analysis of the study group alone revealed significant positive correlations between MMP-9 and TSH (r = 0.74 p = 0.036), BDNF and both ALT (r = 0.73 p = 0.038) and TSH (r = 0.85 p = 0.008), and a negative correlation between MMP-1 and fT4 (r = -0.75 p = 0.032). The control group did not present any significant correlations.

Out of the three patients with excessive BMI in the study group, two (one obese and one overweight) had hypercholesterolemia. No other metabolic disturbances were found.

## Discussion

This cross-sectional study confirmed higher concentrations of BDNF and lower of MMP-2 in non-MetS girls with TS compared to healthy girls with short stature. These findings present a protective trend and may prove important in the context of the “natural” development of the metabolic status in Turner syndrome patients.

Similar to our previous study, we found differences in BDNF concentration between the analyzed groups. In the previous analysis, we also found a higher concentration of BDNF in girls with TS treated with GH compared to healthy short-stature girls (5). In both studies, the absence of correlation between BDNF and IGF-1 was confirmed. It should be noted that in our study, the groups were comparable in chronological and bone age as well as in weight, BMI Z-Score and hsds. The only clinical difference we found between the groups was birth weight. (Previously, we had observed differences in BMI Z-score between groups, which could account for the observed difference; however, in this study, the groups were comparable in terms of BMI Z-score). In both studies we showed a positive correlation between BDNF and BMI Z-score. Of the three highest BDNF values obtained, two were found in an obese and in an overweight patient.

According to the literature, difference in BDNF concentrations between the groups could also relate to estrogen-androgen imbalance in TS. This would be in line with the results of the study performed by Czyzyk et al., who described a two-fold increase in the concentration of BDNF in a group of adult TS patients compared to the controls. In their study, BDNF correlated positively with testosterone levels in TS women, and the authors hypothesized that androgens can be one of the main regulators of plasma BDNF levels (12). Additionally Apter et al. showed that differences in androgen levels are noticeable in TS as early as at the age of 13 (30). However, most of the patients participating in our study were in the pre-pubertal period and we have not conducted relevant studies to support this hypothesis.

Our analysis showed no significant differences between the study group and the control group in the concentration of MMP-1 and MMP-9; however, a difference was observed in the level of MMP-2. Additionally, MMP-2 significantly correlated with HDL concentration: patients with the highest concentration of MMP-2 had a high level of HDL.

Studies in humans have shown that the plasma levels of MMP-2 and MMP-9 are elevated in patients with the metabolic syndrome (22). Our population included only one obese patient in the study group. What is more, short stature girls and patients with TS did not differ in mean baseline concentration of glucose, HDL, and TG levels. We found that the HDL concentration was within the normal range in all the examined girls; fasting glucose concentration was impaired in two girls in the study group; hyper TG was observed in one girl in the study group and in two controls. This might explain the absence of differences in MMP-9 between the analyzed groups. Study group patients with and without impaired carbohydrate-lipid metabolism haven’t differed in MMP-9 concentrations.)

By contrast, no explanation was found for the differences in MMP-2 levels between the analyzed groups. In our study we haven’t noticed a difference in MMP-2 concentrations between girls with and without impaired carbohydrate-lipid parameters. The correlation between HDL and MMP-2 concentrations is difficult to explain. What is more, some results indicate the inhibition of MMP-2 activation as a mechanism for HDL-mediated cardioprotection (31) and suggest negative correlation between HDL and MMP-2 activity (32). However, it is known that the synthesis of MMP-2 is influenced by many factors, which makes the explanation of individual observations a difficult task. The concentration of MMP-2 is also known to correlate with parameters, such as the thickness of the intima-media complex (33) or blood pressure values (34), not analyzed in this study. In order to clarify this difference, a study on a larger group of TS patients, with analysis of additional factors, is needed.

MMP-1 is involved in adipose tissue remodeling in obesity (21). In our previous study we found higher concentrations of this metalloproteinase in the study group, but we also found higher Z-score BMI in the study group and a positive correlation between MMP-1 and Z-score BMI, possibly confirming a link between MMP-1 and body weight (5). However, a larger study performed by Papazoglou et al. in a group of more than 100 obese patients revealed no significant differences in MMP-1 concentrations between obese and lean participants (35). Further research is needed.

GDNF concentration was below the limit of detection. In our previous study, the concentration of GDNF was low, but detectable in plasma. However, we found no differences between the analyzed groups at that time (5). According to some studies, plasma GDNF concentrations seem to poorly reflect its concentrations in tissues which are involved in metabolic regulation (14).

We observed no differences in VEGF concentrations between the analyzed groups. As mentioned above, the available studies on the association of VEGF and metabolic disorders have given contradictory results – some studies revealed higher levels of VEGF in overweight and obese patients (18, 19), whilst other studies proved the opposite (15, 36). In our previous study, we found no difference between the TS group and control group (5), which is further confirmed by this study. It is worth mentioning, however, that many factors affect the concentration of VEGF. This factor is involved in many physiological and pathological conditions such as lymhangiogenesis, embryogenesis, wound healing, inflammation, tumor metastasis, cardiovascular diseases, or rheumatoid arthritis (37). VEGF exerts an influence mainly locally, as in the case of wound healing or atherosclerotic process. Nevertheless, despite the local effect on tissues, its serum concentration is increased in several types of cancers (38) as well as there are significant familial correlations of plasma VEGF concentration between genetically related individuals (39). Given individual variability of VEGF’s circulating levels, more research is needed in this area to clearly establish the factors influencing its concentration in plasma and avoid incorrect associations.

We are aware of the limitations of our study: it was performed on a small number of young patients. This is due to the fact that TS is classified as a rare disease, and it takes time to collect a large number of GH-untreated patients. Future studies could include the correlation between the concentrations of metabolic markers with blood pressure values obtained from Holter RR recordings. It could also be useful to compare the levels of metabolic markers in TS girls and in normal weight, overweight and obese girls.

Despite of the above limitations, the study remains unique in the context of the analyzed group and the selected metabolic markers. Moreover, the presented markers also play roles other than those discussed here. The strength of this work lies in its well selected control group and novel take on biomarkers of metabolic risk. This study is a part of a series of markers determinations in patients with TS. The study performed before the onset of GH therapy allows to exclude its potential influence on carbohydrate and lipid metabolism that could be connected with shifts in markers concentration. It is known that in the TS group, the effect of GH therapy on carbohydrate-lipid parameters was found (40–42). Hence, this research seems to be valuable, because study group was composed of girls who had not been treated with GH so far. In our previous study, the influence of GH on the results could not be ruled out. In addition, we are currently conducting further research on marker stability and repeatability, the impact of rGH, the impact of BMI changes, and the appearance of MetS components.

## Data Availability Statement

The original contributions presented in the study are included in the article/supplementary material. Further inquiries can be directed to the corresponding author.

## Ethics Statement

The studies involving human participants were reviewed and approved by Ethics Committee of the Medical University of Silesia (resolution number KNW/0022/KB1/162/15/16). Written informed consent to participate in this study was provided by the participants’ legal guardian/next of kin.

## Author Contributions

EB and AG designed the study, prepared the database, and wrote the manuscript. JGi monitored the patients and collected samples for biochemical analysis. JGa analyzed the patient database and wrote the manuscript. TF collaborated in designing the work and performed laboratory analyses. MKF and GH performed laboratory analyses. All authors contributed to the article and approved the submitted version.

## Funding

Financial resources were granted as part of financing tasks aimed at the development of doctoral studies and statutory work. The number of research funding contract is KNW-2-K27/D17/N and KNW-1-070/N/9/K (Medical University of Silesia).

## Conflict of Interest

The authors declare that the research was conducted in the absence of any commercial or financial relationships that could be construed as a potential conflict of interest.

## Publisher’s Note

All claims expressed in this article are solely those of the authors and do not necessarily represent those of their affiliated organizations, or those of the publisher, the editors and the reviewers. Any product that may be evaluated in this article, or claim that may be made by its manufacturer, is not guaranteed or endorsed by the publisher.
